# Histone Deacetylase Inhibitors Activate Tristetraprolin Expression through Induction of Early Growth Response Protein 1 (EGR1) in Colorectal Cancer Cells

**DOI:** 10.3390/biom5032035

**Published:** 2015-08-28

**Authors:** Cyril Sobolewski, Sandhya Sanduja, Fernando F. Blanco, Liangyan Hu, Dan A. Dixon

**Affiliations:** Department of Cancer Biology, University of Kansas Medical Center, Kansas City, KS 66160, USA; E-Mails: Cyril.Sobolewski@unige.ch (C.S.); sandhyasanduja@gmail.com (S.S.); Fernando.Blanco@jefferson.edu (F.F.B.); lyhukk@yahoo.com (L.H.)

**Keywords:** HDAC, TTP, post-transcriptional regulation, COX-2, colon cancer

## Abstract

The RNA-binding protein tristetraprolin (TTP) promotes rapid decay of mRNAs bearing 3' UTR AU-rich elements (ARE). In many cancer types, loss of TTP expression is observed allowing for stabilization of ARE-mRNAs and their pathologic overexpression. Here we demonstrate that histone deacetylase (HDAC) inhibitors (Trichostatin A, SAHA and sodium butyrate) promote TTP expression in colorectal cancer cells (HCA-7, HCT-116, Moser and SW480 cells) and cervix carcinoma cells (HeLa). We found that HDAC inhibitors-induced TTP expression, promote the decay of COX-2 mRNA, and inhibit cancer cell proliferation. HDAC inhibitors were found to promote TTP transcription through activation of the transcription factor Early Growth Response protein 1 (EGR1). Altogether, our findings indicate that loss of TTP in tumors occurs through silencing of EGR1 and suggests a therapeutic approach to rescue TTP expression in colorectal cancer.

## 1. Introduction

Colorectal cancer (CRC) is a leading cause of cancer-associated morbidity and mortality among adults worldwide despite existing therapies (surgery, radiotherapy and chemotherapeutic agents) [1]. The onset and progression of this disease results from an accumulation of genetic and epigenetic defects that allow for altered gene expression and overabundance of tumor promoting factors [2]. In normal intestinal epithelium, post-transcriptional mechanisms play a crucial role in regulating the expression of growth-promoting and inflammation-associated gene expression [3]. A conserved feature associated with these genes is the AU-rich element (ARE) present in the mRNA 3' untranslated region (3' UTR). This *cis*-acting regulatory element serves as sites for *trans*-acting RNA-binding proteins that mediate events leading to rapid mRNA decay or stability [4]. During the process of CRC development, the ability of the ARE to function as an mRNA decay element is lost allowing for overexpression of factors that promote tumor growth, invasion, and angiogenesis [5,6,7]. Better understanding of the defects occurring at the post-transcriptional level may provide novel therapeutic approaches, which can improve patient prognosis.

Tristetraprolin (TTP, *ZFP36*) is a well-characterized RNA-binding protein that functions to promote rapid decay of ARE-containing target mRNAs [8]. The physiological significance of TTP in post-transcriptional gene regulation of ARE-mRNAs is evident from the *Zfp36* knockout mouse model, where these mice develop multiple inflammatory syndromes resulting from increased expression of tumor necrosis factor-α (TNF-α), cyclooxygenase-2 (COX-2) and other pro-inflammatory factors due to defects in their respective rapid mRNA turnover [9,10,11,12]. Current work has identified distinct signaling pathways that regulate the expression and function of TTP in different cell types, with regard to its phosphorylation, sub-cellular localization, ARE-binding, and interaction with other cellular proteins [13]. Our recent work examining TTP expression in various stages of colorectal cancer has shown that loss of TTP expression occurs at early stages of tumorigenesis and how ectopic expression of TTP in colon cancer cells attenuates cell proliferation [7]. Through its ability to bind AREs and target the bound mRNA for rapid degradation, TTP can limit the expression of a number of critical genes frequently overexpressed in inflammation and cancer, thereby acting in a tumor suppressor capacity. However, what still remains unanswered is how CRC cells lose TTP expression during the process of carcinogenesis since *ZFP36* (located on 19q13.1) does not appear to be a target of genomic loss or rearrangement in CRC [14].

Epigenetic mechanisms of gene silencing allow for the transcriptional repression of tumor suppressor genes in various human malignancies including CRC [15]. DNA methyltransferases (DNMTs) catalyze DNA methylation at CpG islands, typically in promoter regions of the genome, and negatively impact transcription [16]. Chemical modifications of histones, most commonly acetylation of histone H3 and H4 reduce the affinity between histones and DNA, allowing for promoter region accessibility to transcription factors and RNA polymerase [17]. However, histone deacetylases (HDACs) promote transcriptional repression and have been associated with silencing of many tumor suppressor genes, since many HDACs are overexpressed in cancer [15,18]. Consistent with this, HDAC inhibitors provide an efficient pharmacologic approach for cancer therapy by restoring tumor suppressor gene expression [15].

In this study, we investigated if HDAC inhibitors can restore TTP expression in CRC cells. Our results show that HDAC inhibitors induce TTP expression at the transcriptional level, leading to the downregulation of the ARE-containing gene, COX-2. Further analysis indicated an indirect epigenetic regulation of TTP was occurring and TTP induction was owing to an upregulation of EGR1, a known transcription factor for TTP. These findings bring new insights into loss of post-transcriptional regulatory circuits during CRC tumor development and demonstrate that HDAC inhibitors could restore TTP expression in cancer cells, thus providing clinical perspectives for these compounds.

## 2. Results

### 2.1. HDAC Inhibitors Promote TTP Expression in CRC Cells

**Figure 1:**
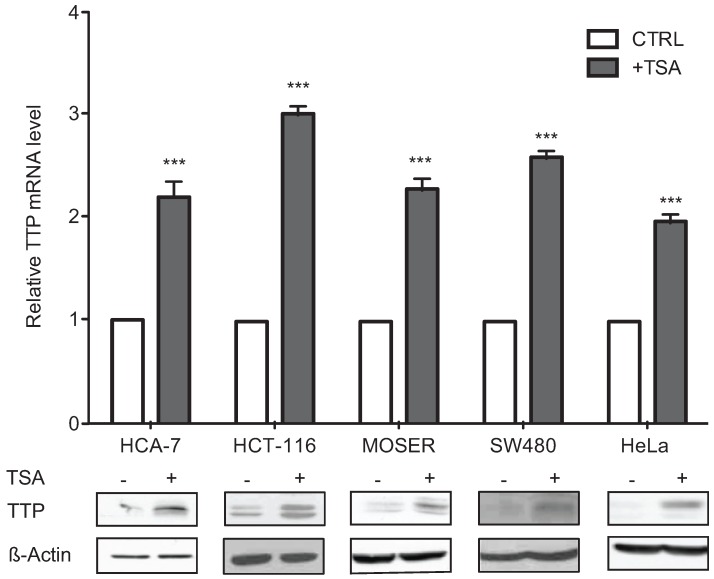
HDAC inhibitors promote TTP expression. Colon cancer (HCA-7, HCT116, Moser, SW480) and HeLa cells treated with 0.4 μM TSA for 12 h were examined for changes in TTP mRNA levels (**top**) by qPCR using GAPDH as a loading control and normalized to non-treated cells. TTP protein (**bottom**) expression was analyzed by Western blot using β-actin as a loading control. The data represent the mean of three independent experiments (± SD). *** *p* < 0.001.

Our work and that of others have shown loss of TTP expression to occur in colon cancer cells and tumors as compared to normal tissue [7,19,20], however the mechanisms of TTP loss are not known. To determine if chromatin remodeling was involved in *ZFP36* gene silencing, CRC cells (HCA-7, HCT116, Moser, and SW480) and the cervical cancer cell line HeLa were treated with trichostatin A (TSA) followed by analysis of TTP mRNA and protein expression. In all cell lines examined TSA treatment promoted a 2- to 4-fold induction in TTP mRNA and protein (Figure 1).

**Figure 2:**
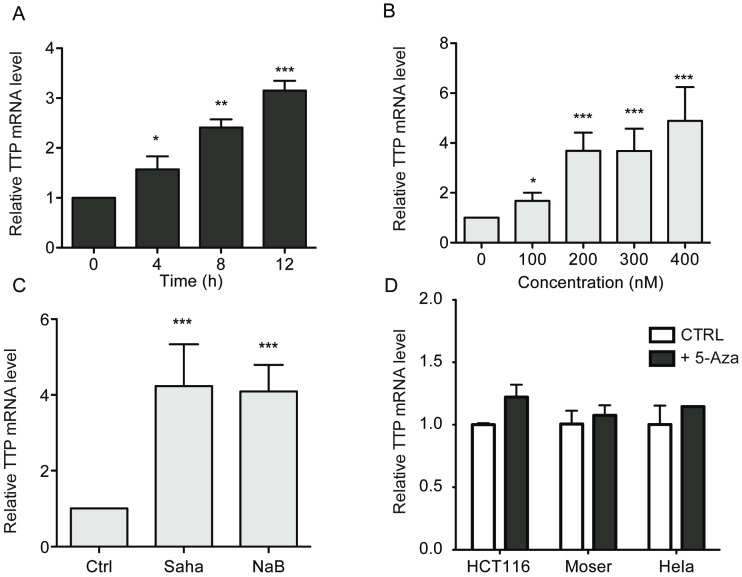
HDAC inhibitors promote TTP expression. (**A**) Time course of TTP mRNA expression in HCT116 cells treated with 0.4 μM TSA. (**B**) TTP induction in HCT116 cells treated cells treated with different doses of TSA for 12 h. (**C**) HCT116 cells were treated for 12 h with 5 μM SAHA or 5 mM sodium butyrate (NaB) and assayed for TTP mRNA expression by qPCR. (**D**) Cells (HCT116, Moser, and HeLa) untreated or treated with 10 μM 5-Aza-dC for 48 h were examined for TTP mRNA levels by quantitative PCR using GAPDH as normalization control. The data represent the mean of three independent experiments (± SD). * *p* < 0.05, ** *p* < 0.01, *** *p* < 0.001.

In HCT116 cells, TSA-mediated induction of TTP was time- and dose-dependent manner (Figure 2A,B). Similar results were obtained with using the HDAC inhibitors SAHA and sodium butyrate (NaB) (Figure 2C), further indicating the involvement of HDACs in *ZFP36* silencing. Previous reports have shown that the loss of TTP can be mediated by CpG island methylation within TTP promoter [21]. However, we did not found any alteration of TTP expression in HCT-116, Moser and HeLa cells treated with the DNA demethylating agent 5-Aza-2'-deoxycytidine (5-Aza-dC) (Figure 2D).

### 2.2. TSA-Induced TTP Expression Contributes to ARE-mRNA Decay and Growth Inhibition

**Figure 3:**
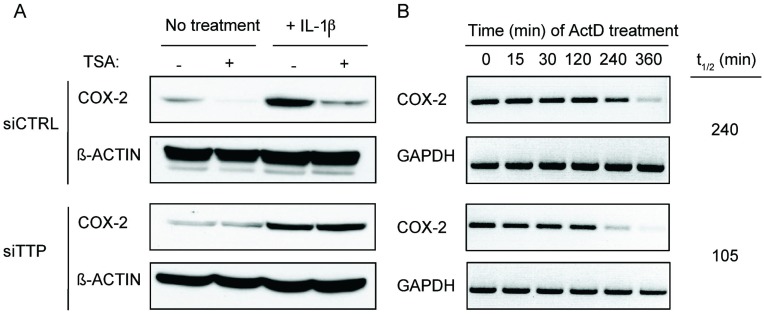
TSA-induced expression of TTP promotes ARE-mRNA decay. (**A**) HeLa cells were treated left untreated or stimulated with 10 ng/mL of IL-1β for 24 h to induce COX-2 expression. Cells were then transfected with control or TTP siRNA for 36 h and then treated with TSA (0.4 μM) for 12 h. COX-2 and β-actin were analyzed by Western blot. (**B**) HeLa cells were stimulated with IL-1β (10 ng/mL) for 24 h and then treated with 0.4 μM TSA for 12 h. Act D (5 μg/mL) was added for the indicated time points, after which COX-2 mRNA stability was assayed by RT-PCR using GAPDH as a loading control. Blots shown are representative of duplicate experiments.

Based on its ability to promote TTP expression, we hypothesized that TSA treatment would inhibit ARE-mRNA expression. To test this, TSA-treated HeLa cells were examined for respective changes in the ARE-containing TTP-target mRNA COX-2 [7]. HeLa cells represent a suitable model to study COX-2 expression, since its expression can be induced by pro-inflammatory cytokines such as IL-1β (Figure 3A). In both non-treated and IL-1β-stimulated cells, TSA-treatment attenuated COX-2 protein levels compared to their respective non-treated controls (Figure 3A, top). This effect was dependent on TSA-mediated induction of TTP since downregulation of TTP by siRNA abrogated COX-2 downregulation in TSA-treated cells (Figure 3A, bottom). To determine if TSA inhibited COX-2 expression by promoting mRNA decay, IL-1β-stimulated HeLa cells were treated with TSA for 12 h and COX-2 mRNA half-life was assessed after actinomycin D (ActD) was added to cells to halt transcription. Figure 3B shows that TSA promoted enhanced decay of COX-2 mRNA (t_1/2_ = 105 min) as compared to control-treated cells (t_1/2_ = 240 min).

**Figure 4:**
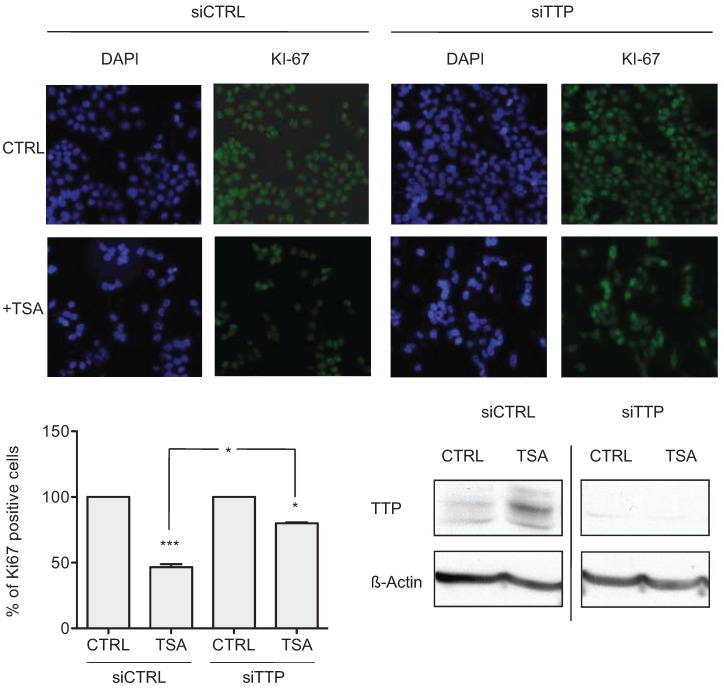
TSA-induced expression of TTP promotes growth inhibition. Immunofluorescent detection of Ki-67, shown in green, in HCT116 cells transfected with control siRNA or TTP siRNA for 24 h and then treated with 0.4 μM TSA for additional 24 h. DAPI nuclear staining is shown. Bar graphs represent the average percentage of Ki-67 positive cells per field ± SD (*n* = 3 fields) of 3 independent experiments. The efficiency of TTP knockdown is shown by Western blot of TTP expression after 24 h of siRNA transfection followed by 12 h of TSA treatment. The data represent the mean of three independent experiments (± SD). * *p* < 0.05, *** *p* < 0.001.

The ability of HDAC inhibitors to alter CRC cell growth is well established [15]. To determine if this effect is mediated by induction of TTP by HDAC inhibitors, siRNA-knockdown of TTP was performed in HCT116 cells treated with TSA and evaluated for proliferation by Ki-67 staining (Figure 4). With the control siRNA, TSA-treatment decreased the number of Ki-67 positive cells by 53.36 ± 1.66%. Whereas in TTP deficient cells, TSA decreased the number of Ki-67 positive cells only 19.92 ± 0.52%. These results are consistent with TTP-dependent growth inhibition of CRC cells observed through adenoviral delivery of TTP [7] indicating that TTP contributes to TSA-induced growth inhibition.

### 2.3. HDAC Inhibitors Promote TTP Transcription

**Figure 5:**
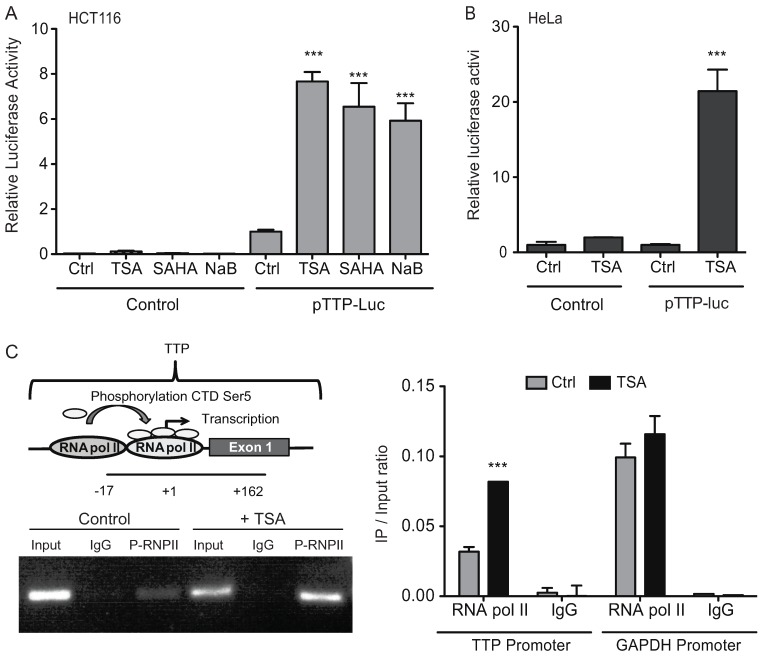
HDAC inhibitors increase TTP transcription. (**A** and **B**) HCT116 and HeLa cells were transfected with a luciferase reporter containing 659 bp of the human TTP promoter (pTTP-Luc) or control pGL3-Basic for 36 h, and treated with 0.4 μM TSA, 5 μM SAHA, or 5 mM NaB for 12 h. Luciferase activity was normalized to total protein. Values shown are normalized to Luc expression in the respective control-treated (Ctrl) cells and indicate the average of 3 independent experiments ± SD. (**C**) ChIP of phosphorylated RNA Pol II bound to TTP promoter. Sonicated chromatin from HCT-116 cells treated with 0.4 μM TSA for 12 h was immunoprecipitated with anti-RNA pol II phospho-CTD (Ser5) or nonspecific IgG. PCR analysis of eluted DNA was performed using primers to amplify a 179 bp region (−17/+162) of the *ZFP36* promoter. 0.5% of input DNA is shown in ethidium bromide-stained gels. qPCR was used to quantitate fold-enrichment of *ZFP36* promoter (−17/+162) or control *GAPDH* promoter in respective ChIP reactions. The data are represented as the immunoprecipitated (IP) and input chromatin signal ratio and are the average of 3 experiments ± SD. *** *p* < 0.001.

In order to determine that induction of TTP in response to HDAC inhibitor treatment reflects an actual transcriptional event, we examined the ability of TSA to influence TTP promoter activity. A luciferase expression construct containing 659 bp of the human TTP promoter [21] was transfected into HCT116 cells and treated with TSA, SAHA, or NaB. Shown in Figure 5A, treatment of cells with HDAC inhibitors resulted in a significant increase in TTP promoter activity indicating their ability to promote *ZFP36* transcription. Similar results were observed in TSA-treated HeLa cells (Figure 5B). As a measure of transcriptional activation by TSA, the presence of activated RNA polymerase II at the endogenous *ZFP36* promoter was evaluated by chromatin immunoprecipitation (ChIP) (Figure 5C). ChIP assay was performed using an antibody against RNA polymerase II phosphorylated on Ser5 of the C-terminal domain (CTD) as a marker for active gene transcription, followed by PCR to detect binding within the −17/+162 *ZFP36* promoter region. Compared to control-treated cells, TSA promoted a >2-fold increase in association of RNA pol II phospho-CTD to the TTP promoter (Figure 5C), which is consistent with the level of induction of TTP mRNA by TSA treatment (Figure 1). This enrichment was not observed at the control *GAPDH* promoter.

**Figure 6:**
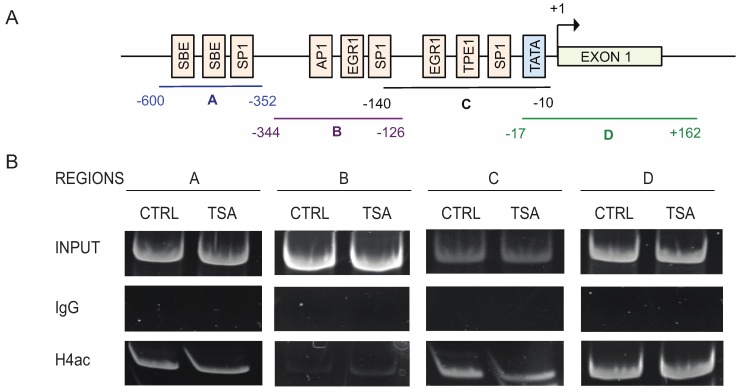
TSA does not impact TTP promoter histone acetylation. (**A**) Schematic representation of the *ZFP36* promoter. Transcription factors binding sites and the different regions tested for ChIP (A, B, C, and D) are shown. (**B**) ChIP of acetylated histone H4 in the *ZFP36* promoter. Chromatin from HCT-116 cells treated with 0.4 μM TSA for 12 h was immunoprecipitated with anti-acetylated histone H4 or nonspecific IgG. PCR analysis of eluted DNA was performed using primers spanning regions A, B, C, and D of *ZFP36* promoter as described in Experimental Section. Gels shown are representative of duplicate experiments.

A primary mechanism by which HDAC inhibitors promote gene expression is through increased acetylation status of histones, with acetylation of histone H3 and H4 in promoter regions commonly associated with activated gene expression [15]. To determine if TSA promoted TTP expression through this mechanism, we analyzed the acetylation status of histone H4 in ~700 bp spanning the *ZFP36* promoter in HCT116 cells by ChIP (Figure 6). Using a pan-acetylated histone H4 antibody, acetylation of histone H4 was observed at varying levels within *ZFP36* promoter. However, treatment with TSA did not further increase H4 acetylation in the respective regions. These results indicate the ability of TSA to activate TTP transcription was not a direct effect of chromatin remodeling on *ZFP36* promoter and suggested TSA acted through an alternative mechanisms possibly promoting expression of transcription factors regulating TTP.

### 2.4. Early Growth Response Protein 1 (EGR1) is Associated in TSA-Induced TTP Expression

**Figure 7:**
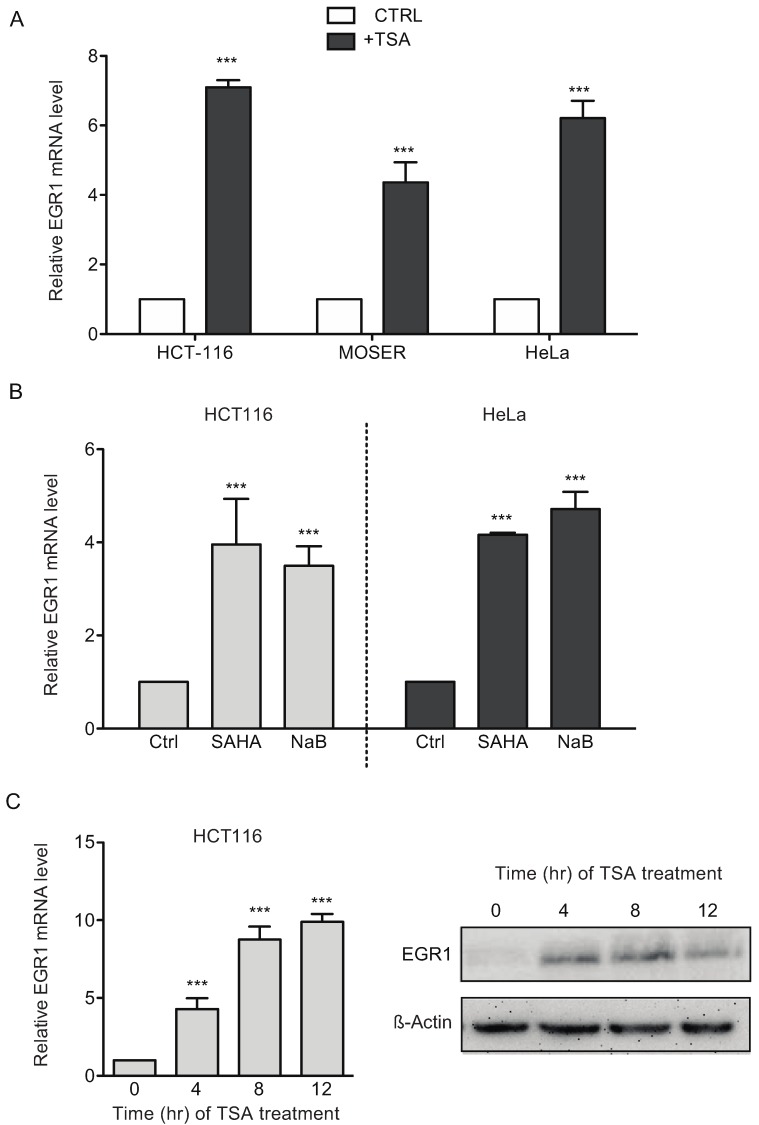
HDAC inhibitors induce EGR1 expression. (**A**) HCT116, Moser and HeLa cells were treated with 0.4 μM of TSA for 12 h and then examined for changes in EGR1 mRNA levels by qPCR. (**B**) The expression of EGR1 in HCT116 and HeLa cells treated for 12 h with SAHA (5 μM) or NaB (5 mM) by qPCR analysis. (**C**) Time course (0, 4, 8 and 12 h) of EGR1 expression in HCT116 cells treated with TSA (0.4 μM). The expression of EGR1 was analyzed by qPCR (**left**) and western blot (**right**). qPCR was performed using GAPDH as a loading control. The data represent the mean of three independent experiments (± SD). *** *p* < 0.001.

Various transcription factors control the transcription of TTP [22,23]. Recently, the role of EGR1 has been highlighted in breast cancer cell model and HDAC inhibitors can induce this transcription factor [24,25]. EGR1 is also associated with an immediate/early gene expression response, consistent with the early induction of TTP observed upon TSA treatment (Figure 2). Therefore, we hypothesized that EGR1 might be implicated in TSA-induced TTP expression. We found that TSA treatment promoted a 4- to 7-fold induction of EGR1 mRNA expression in HCT116, Moser and HeLa cells (Figure 7A). We observed similar results in HCT116 treated with SAHA and NaB (Figure 7B). Our data show also that this induction can be observed at the mRNA and at the protein level and in a time-dependent-manner (Figure 7C), similar to TTP induction.

**Figure 8:**
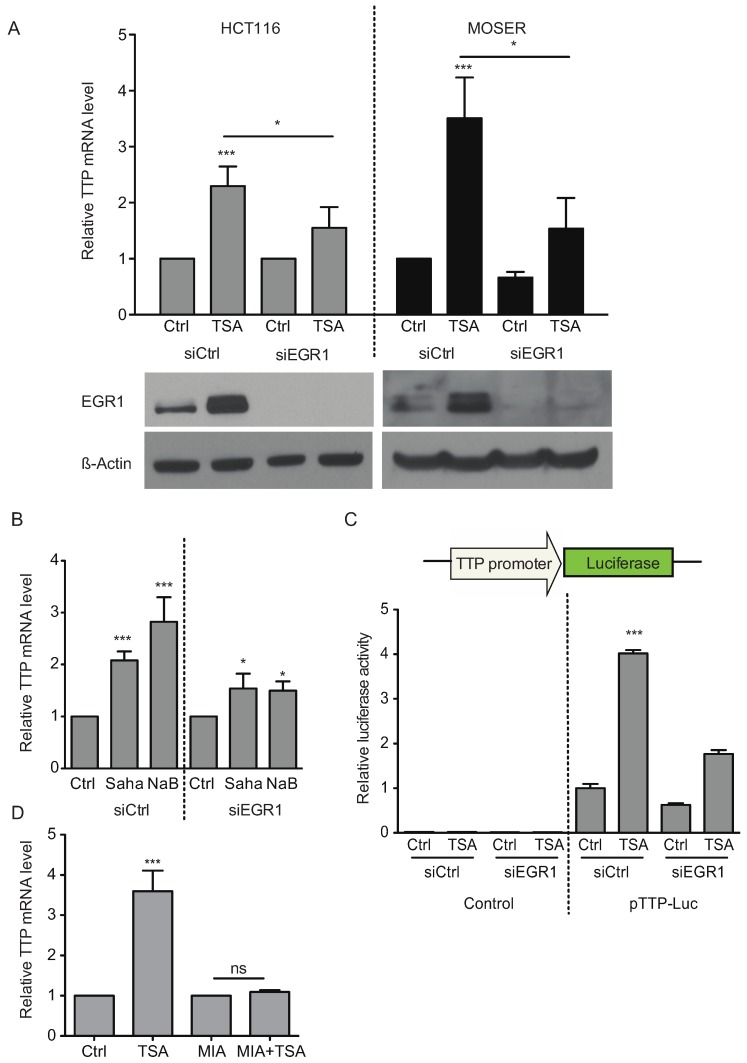
HDAC inhibitors induce TTP expression partially through EGR1. (**A**) HCT116 or Moser cells were transfected with 100 nM of control siRNA or siRNA against EGR1 for 24 h. Then the cells were treated with TSA (0.4 μM) for 12 h. The expression of EGR1 was analyzed by Western blot and TTP expression was assayed by qPCR. (**B**) A similar experiment was performed in HCT116 cells treated with SAHA (5 μM) or NaB (5 mM). (**C**) HCT116 cells were transfected with pTTP-Luc or control pGL3-Basic and a control siRNA or siRNA against EGR1 for 24 h. Cells were then treated for 12 h with TSA (0.4 μM) and luciferase activity was measured. (**D**) HCT116 cells were treated 24 h with 500 nM of mithramycin A prior to 12 h of treatment with TSA (0.4 μM). Expression of TTP was assayed by qPCR. Data represent the mean of three independent experiments (± SD). * *p* < 0.05, ** *p* < 0.01, *** *p* < 0.001.

We also found that EGR1 is partially responsible for TSA-induced TTP expression. Silencing of EGR1 by siRNA partially attenuated TSA-induced TTP expression, with a significant reduction observed in HCT116 and Moser cells (Figure 8A). Similar results were obtained with SAHA and NaB treatment (Figure 8B). Furthermore, TSA-induced TTP promoter activity was similarly reduced in EGR1 knockdown cells (Figure 8C). EGR1 is a zinc-finger protein known to bind GC-rich domains in the DNA (5'-GNG TGG GCG-3') [26]. The pre-treatment of HCT116 cells with the GC-rich DNA-binding inhibitor, mithramycin A, completely reverted TSA-induced TTP expression (Figure 8D), thus providing an additional evidence connecting EGR1 in TTP induction.

**Figure 9:**
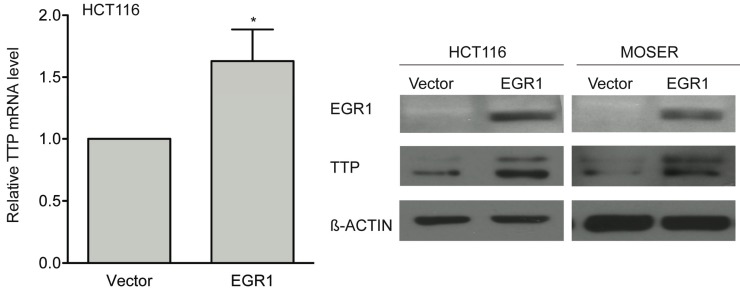
EGR1 expression induces TTP expression. HCT116 and Moser cells were transfected with 1 μg of pcDNA3.1/Zeo/EGR1-Flag or vector control. Twenty-four hours post-transfection, cells were harvested for RNA and protein analysis. EGR1 expression was assayed by Western blot (right panel). TTP expression was assayed by qPCR and Western blot. The data are the mean ± SD of three independent experiments. * *p* < 0.05.

To determine if EGR1 could promote TTP expression independent of HDAC inhibition, an EGR1 expression construct was transfected into HCT116 and Moser cells. Shown in Figure 9, exogenous expression of EGR1 promoted increased TTP expression at the mRNA and at the protein level. These findings indicate that TTP induction depends on restoration EGR1 expression, rather than epigenetic event(s) occurring at the TTP promoter.

**Figure 10:**
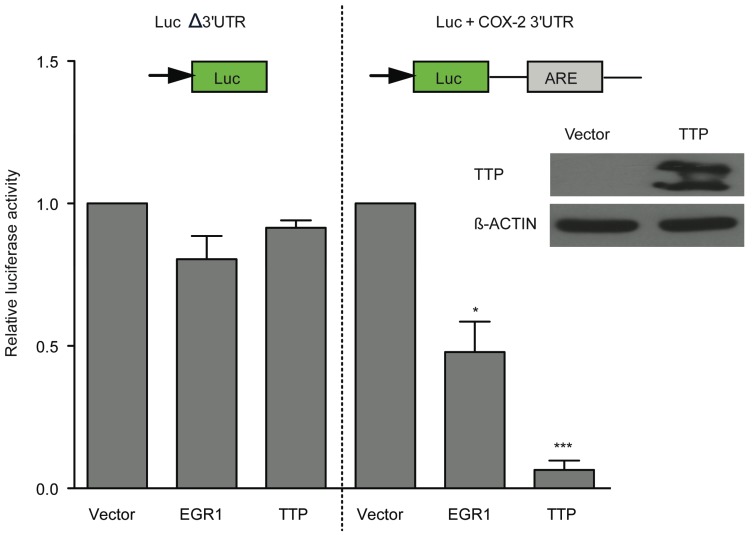
EGR1 expression downregulates COX-2 in an ARE-dependent manner. HCT116 cells were transfected with pcDNA3.1/Zeo/EGR1-Flag or vector control, along with luciferase reporters with (Luc+COX-2 3' UTR) or without (LucΔ3' UTR) the 3' UTR region of COX-2. Twenty-four hours post-transfection, luciferase activity was assayed and normalized to total protein concentration. As a positive control, transfection of a TTP expression construct was performed; inset shows TTP protein expression in transfected cells by Western blot. The data are the mean ± SD of three independent experiments. * *p* < 0.05, *** *p* < 0.001.

As EGR1 expression promoted TTP expression in HCT116 cells, we next tested if EGR1 overexpression could influence COX-2 mRNA decay. To examine this, luciferase reporter constructs containing the COX-2 3' UTR (Luc+COX-2 3' UTR) or control luciferase (LucΔ3' UTR) [27] were co-transfected with an empty vector or EGR1 expression construct in HCT116 cells. Figure 10 shows that in presence of EGR1, luciferase activity from the Luc+COX-2 3' UTR reporter is reduced approximately 2-fold. As a positive control, transfection of a TTP expression construct in HCT116 cells yielded a >90% reduction of Luc+COX-2 3' UTR activity. These data suggest that EGR1 is implicated in mRNA decay of transcripts bearing a 3' UTR ARE.

### 2.5. P-Bodies, TTP and EGR1 Are Decreased in Colorectal Tumors

**Figure 11:**
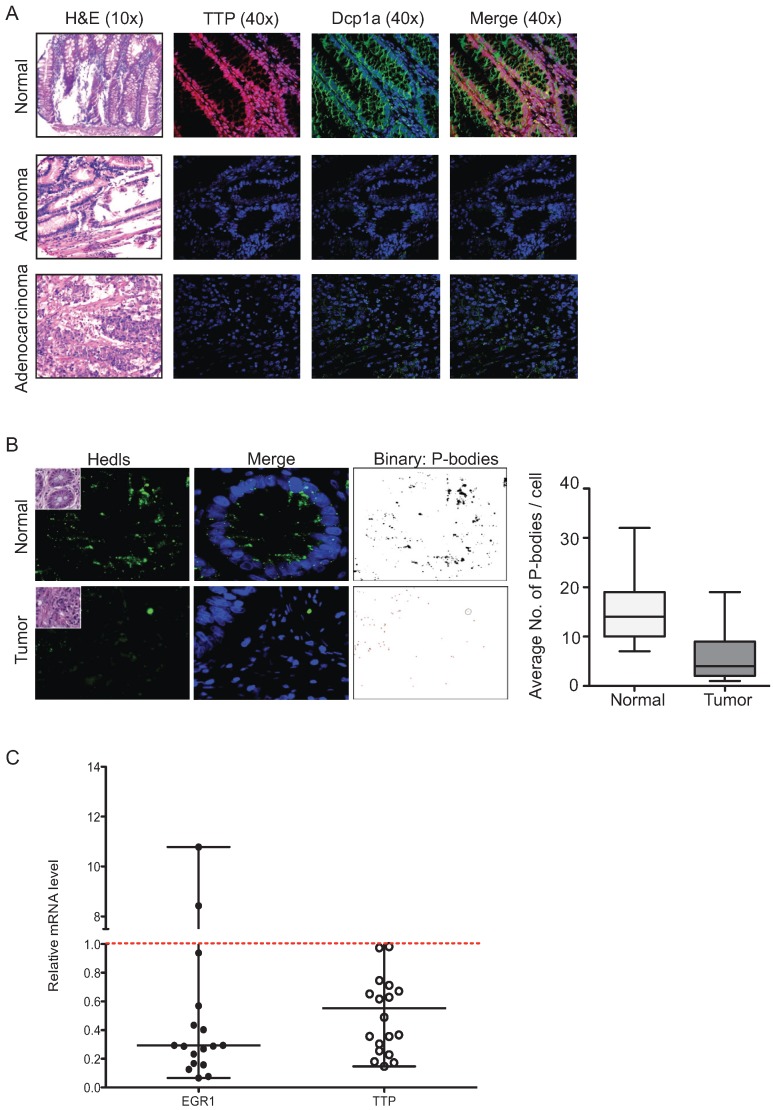
TTP loss in tumor tissue correlates with P-bodies loss and EGR1 downregulation. (**A**) Hematoxylin/eosin staining and immunofluorescence of TTP, Dcp1a on normal human colon tissue, adenoma, and adenocarcinoma. Staining shown is representative of CRC tissue array samples described in Experimental Section. (**B**) Immunofluorescence staining of P-bodies in normal colon and tumor tissue, using anti-Hedls antibody. P-bodies were quantified by ImageJ software by using images obtained from 14 normal and 20 tumor samples and quantitated in binary mode. (**C**) Expression of EGR1 (*n* = 17) and TTP (*n* = 18) mRNA was examined in total RNA isolated from human colon tumors and normal colon tissue by qPCR. GAPDH was used as a loading control. Relative EGR1 and TTP expression levels for tumors were normalized to their respective matched normal tissue.

ARE-mediated mRNA decay requires the involvement of many enzymes required for the deadenylation (Pan2-Pan3 complex), decapping (e.g., Dcp1a, Hedls) and RNA degradation (e.g., XRN1) of the target mRNA. This machinery is localized in small cytoplasmic foci processing (P)-bodies [28,29]. Our recent findings revealed that TTP is an important determinant for P-body assembly in normal intestinal epithelium [30]. In order to determine whether TTP loss in CRC is associated with P-bodies alteration, we analyzed the expression of TTP and the P-body marker Dcp1a in normal human colon, adenoma, and adenocarcinoma FFPE tissue arrays (Figure 11A). We found the expression of both TTP and P-bodies were strongly reduced in tumor tissue. In addition, we performed immunofluorescence analysis for P-bodies using Hedls as a marker in human colon tissues and we found that the number of P-bodies is significantly reduced in tumor tissue (Figure 11B), indicating that TTP loss is associated with decreased P-bodies in CRC tumors. Regarding the causal link existing between TTP and EGR1 expression, we hypothesized that EGR1 is also downregulated in CRC tissues. This was evaluated by assaying TTP and EGR1 expression in normal colon and tumor tissue by qPCR (Figure 11C). Both TTP and EGR1 were significantly downregulated in 90% of tumor tissues compared to normal tissue (EGR1: *p* = 0.0277; TTP: *p* = 0.0065). A calculation of the Pearson correlation coefficient between EGR1 and TTP expression in normal and tumor tissues gave a value of *r =* 0.84 indicating a high positive correlation. This data suggests that the loss of EGR1 expression in CRC could contribute to the loss of TTP expression, which in turn could affect the number of P-bodies. 

## 3. Discussion

TTP negatively regulates cell growth in several cancer cell types by inhibiting the stability of many mRNA implicated in cell cycle (c-Myc, cyclin D1), inflammation (TNFα, COX-2), apoptosis (Bcl-2, Mcl-1), and angiogenesis (VEGF) [7,13,31,32]. Even though several lines of evidence have established loss of TTP as a conserved feature in colorectal cancer, the underlying mechanism remains elusive. While no *ZFP36* mutations have been reported as direct genetic link to cancer incidence, specific *ZFP36* polymorphisms are associated with breast cancer survival [33]. In colon cancer, TTP is lost early in tumorigenesis [7], allowing for pathologic overexpression of target genes [3]. In this study, we report that different HDAC inhibitors (TSA, SAHA and sodium butyrate) induce TTP expression in colon and cervical cancer cells. We found that silencing of TTP by siRNA could partially revert TSA-induced growth inhibition, indicating that TTP induction contributes to the growth inhibitory effect of HDAC inhibitors. These data are in line with our previous studies showing that TTP is important for controlling cell proliferation in HeLa cells [34] and colon cancer cells [7]. We further show that TTP induction regulates expression of the ARE-containing COX-2 mRNA, which has previously been shown to contribute to tumor growth [35] and to be a target of TTP-mediated ARE-mRNA decay [7].

The epigenetic regulation of TTP is undefined, leaving a blank area in understanding how TTP is lost in colon cancer. Epigenetic silencing of TTP may occur through direct modulation of the *ZFP36* gene or by silencing transcription factors regulating TTP. Recently, it has been shown that increased DNA methylation of a single CpG site in the *ZFP36* promoter in hepatocellular carcinoma (HCC) is responsible for TTP loss [21]. However, treatment of our cell models with the DNMT inhibitor 5-Aza-dC did not induce TTP expression, suggesting that this mode of epigenetic repression does not appear to play a role in *ZFP36* silencing in colon cancer. In this study, we found that HDAC inhibitor-induced TTP expression was a consequence of an increase in the TTP promoter activity. Interestingly, our findings showed that TSA did not impact histone H4 acetylation within the TTP promoter. It should be noted that our study examined the −600/+162 region of the *ZFP36* promoter, thus other histones modifications or epigenetic alterations in upstream parts of the *ZFP36* promoter may be responsive to HDAC inhibitor treatment. Our findings suggest that the epigenetic events leading to TTP upregulation by HDAC inhibition do not occur on the TTP promoter itself, but at a different locus, e.g., a transcription factor(s) that regulates expression of TTP. In this context, EGR1 represents a good candidate based on its ability to increase TTP expression in breast cancer cells [24] and HDAC inhibitors can induce EGR1 expression [25]. EGR1 is a Cys2His2 zinc finger transcription factor, which binds GC rich region (5'-GNG TGG GCG-3') [26]. In colon cancer, it has been suggested that EGR1 serve as a tumor suppressor [36,37] that is downregulated or lost as a consequence of APC mutation and the subsequent nuclear accumulation of β-catenin [37,38,39]. This current data provides a correlation between EGR1 and TTP loss, however direct evidence linking these two events has not been examined in colorectal cancer. Our data now show that EGR1 is downregulated in colon tumor tissue consistent with loss of TTP expression. Moreover, we found that HDAC inhibitors induce EGR1 expression and the silencing of EGR1 partially reverted TSA-induced TTP expression. Additionally, EGR1 overexpression was sufficient to induce TTP expression and COX-2 mRNA decay. Our data showing that EGR1 knockdown did not completely revert TTP induction suggests that other mechanisms are involved. HDAC inhibitors may affect the expression/activity of other transcription factors known to regulate *ZFP36* transcription such as Smad3, Smad4, AP1, c-myc or NFκB [23,31,40,41] but also some microRNAs, such as miR-29a, which downregulates TTP in a breast cancer model [42] and is known to be upregulated in colon cancer [43]. Consistent with our findings, recent work by Sharma *et al.* [44] has shown that TSA-induced TTP expression leads to the downregulation of claudin-1 in SW480 colon cancer cells. Their results also demonstrated that TSA treatment allowed for increased the binding of Sp1 to the claudin-1 promoter, suggesting that the TSA-dependent induction of Sp1 inhibits claudin-1 transcription. Interestingly, Sp1 and EGR1 have been shown to compete for their binding site on the promoter of several genes [45] and based on their close proximity within the ZFP36 promoter (Figure 6A), this suggests that the ability of HDAC inhibitors to induce TTP expression can be mediated by distinct and/or interconnected mechanisms.

The mechanism(s) by which HDAC inhibitors induce EGR1 expression and activity was not investigated in this study. Previous work has shown that HDAC inhibitors can reactivate EGR1 in various cell types, leading to decreased cell proliferation and increased cell apoptosis [25,46,47,48]. Other mechanisms controlling EGR1 include various signaling pathways such as the EGF (Epidermal Growth Factor)/ELK1 (Ets domain containing protein) pathway [24]. EGR1 can also be regulated on a post-transcriptional level. EGR1 translation is inhibited by miR-183 in different cancer cells, including HCT116 [37]. Further studies connecting these links between EGR1 with the loss of TTP in colon cancer is currently under investigation.

The enzymatic activities of histone deacetylases and histone acetyl transferases (HATs) control the fine dynamics of chromatin structure and gene activation [18,49,50,51]. HDAC inhibitors function by inhibiting different HDAC classes [15]. TSA, SAHA and sodium butyrate inhibit class I (HDACs 1, 2, 3 and 8) and class II (HDACs 4, 5, 6, 7, 9 and 10). In this study, we did not identify which HDACs are implicated in the silencing of EGR1. Many HDACs are known to be upregulated in colon cancer [15]. For example, HDAC2 is upregulated as a consequence of APC mutation and nuclear β-catenin, leading to trans-activation of *HDAC2* promoter [52]. However, silencing HDAC2 did not affect TTP expression (unpublished observations), suggesting that other HDACs are implicated. HDACs also have non-histone substrates, such as transcription factors (e.g., NFκB, Sp1, p53 or EGR1) whose acetylation can affect their transcriptional activity [15,25]. This raises the possibility that TSA-induced TTP expression can be influenced through post-translational modifications on EGR1 protein.

TTP is an important determinant for P-bodies assembly and the loss of TTP is associated with a reduction of the number of P-bodies and thus an impairment of ARE-mediated mRNA decay [30]. In this study, we provide evidence that loss of P-bodies occurs in colon tumors, which is consistent with a concurrent loss of EGR1 and TTP. However, further mechanistic studies are needed in order to better understand the connections between EGR1 and P-bodies. Our data indicating a link existing between EGR1 and TTP suggest that the loss of EGR1 may be a novel contributing factor influencing P-body assembly in colon tumors.

## 4. Experimental Section

*Cell Culture, DNA and siRNA Transfections.* Human colon cancer cell lines HCA7, HCT116, HT29, Moser, and SW480 and cervical cancer cell line HeLa were obtained and cultured as described [27]. Cells were treated for indicated times and concentrations of trichostatin A (TSA), SAHA, Sodium butyrate (NaB), 5-Aza-2'-deoxycytidine (5-Aza-dC), mithramycin A or DMSO control (Sigma Aldrich, St. Louis, MO, USA). In HeLa cells, COX-2 expression was induced by pre-treatment with recombinant human 10 ng/mL Il-1β (R & D systems, Minneapolis, MN, USA) for 24 h where indicated.

Transient transfections of cells with luciferase reporter constructs containing 659 bp of the human *ZPF36* promoter [21] or the COX-2 3' UTR (Luc+3' UTR) [53] were accomplished using Lipofectamine Plus (Invitrogen, Carlsbad, CA, USA) according to the manufacturer’s protocol. Cells were transfected in DMEM for 3 h after which cells were grown in complete medium for 48 h. Transfected cells were lysed in reporter lysis buffer (Promega) and assayed for luciferase activity (Glomax 20/20 Luminometer). Luciferase reporter gene activities were normalized to respective total protein concentrations and assays were done in triplicates. EGR1 expression was accomplished by transfecting cells with pcDNA3.1/Zeo containing the human EGR1 coding sequence with an N-terminal Flag epitope (pcDNA3.1/Zeo/EGR1-Flag) or empty vector. siRNA transfection of cells using 50 nM predesigned siRNAs for human TTP, EGR1, or negative control #1 siRNA (Applied Biosystems,, Foster City, CA, USA) were performed using siQuest (Mirus, Madison, WI, USA) for 48 h according to the manufacturer’s instructions.

*Messenger RNA Analysis.* Total RNA was extracted from cells using Trizol reagent (Invitrogen) according to the manufacturer’s protocol. Complementary DNA (cDNA) synthesis was performed using 1 μg of total RNA in combination with oligo(dT) and Improm-II reverse transcriptase (Promega). qPCR analyses were performed using the StepOnePlus Real-Time PCR Assay System (Applied Biosystems) with TaqMan probes for human TTP (*ZFP36*) or control GAPDH (Applied Biosystems) or gene-specific primers for SYBR green-based assay for EGR1 (sense: 5'-GCC TGCGACATCTGTGGAA-3'; antisense: 5'-GCCGCAAGTGGATCTTGGTA-3'), GAPDH (sense: 5'-CAATGACCCCTTCATTGACC-3'; antisense: 5'-GACAAGCTTCCCGTTCTCAG-3') (Integrated DNA Technology, Coralville, IA, USA). Results were expressed as a ratio: mRNA of target gene/GAPDH mRNA. For COX-2 mRNA stability analysis, a time course analysis was performed using the transcription inhibitor actinomycin D (5 μg/mL) treated with TSA (0.4 μM) for 12 h. The half-life of COX-2 mRNA was calculated by densitometry analysis of the PCR product in an ethidium bromide-stained gel. The values were normalized to GAPDH.

Human colon tumors and histologically normal tissue were obtained from surgical remnants from patients with colorectal cancer through the University of South Carolina Center for Colon Cancer Research Tissue Bank. The protocol was approved by the Institutional Review Board of the University of South Carolina. Tissue was snap-frozen in liquid nitrogen, and total RNA was isolated using TRIzol from approximately 50 mg of tissue.

*Western Blot Analysis.* Cells were lysed in SDS-PAGE lysis buffer (50 mM Tris-HCl, pH 6.8, 100 mM DTT, 2% SDS, 0.1% bromophenol blue, 10% glycerol) and protein content was determined using a BCA protein assay using BSA (bovine serum albumin) as standard (Pierce Biotechnology, Thermo Scientific, Waltham, MA, USA). Lysates (between 20 and 50 μg) were separated by 10% SDS-PAGE, transferred to PVDF membranes (Bio-Rad, Hercules, CA, USA), and blocked for 1 h at room temperature in 5% milk in PBS-Tween. Membranes were probed with antibodies against TTP (38303, Aviva) or EGR1 (4153, Cell Signaling), overnight at 4 °C, and reprobed for 1 h at RT for β-Actin (Clone C4, MP Biomedicals, Santa Ana, CA, USA). Detection and quantitation of blots were performed as described previously [53].

*Chromatin Immunoprecipitation (ChIP).* ChIP assay was performed using ChIP-IT™ Express Chromatin Immunoprecipitation Kit (Active Motif, Carlsbad, CA, USA) according to manufacturer’s instructions. Cells untreated or treated with TSA were subjected to formaldehyde fixation to crosslink DNA-protein complexes and fixed chromatin was sheared by sonication followed by incubation with ChIP validated anti-RNA pol II CTD phospho Ser5 antibody, anti-acetylated histone H4 (SA Biosciences, Valencia, CA, USA) or isotype-matched control IgG. Chromatin was eluted from antibody-bound protein/DNA complexes and reverse cross-linked, and the purified DNA was analyzed by PCR using primers specific for different regions of *ZFP36* promote*r*: Region A sense: 5'-TGTGATCCTCCCAACCCTCT-3', Region A antisense: 5'-GTGCGCGTGCGACACAGAC-3', Region B sense: 5'-GGGCTTCTGCTCTTGTCAAT-3', Region B antisense: 5'-CCCTGGACTGGTTCCCTT-3'; Region C sense: 5'-GGAAGGGAACCAGTCCAG-3', Region C antisense: 5'-ACCGAGAGCCGGCTACTTAT-3'; Region D sense: 5'-CTCTCGGTGCCAGCCTCAG-3', Region D antisense: 5'-CTGGAGTTTGCGGCGCTAGA-3' and *GAPDH* (SA Biosciences). For agarose gel-based PCR analysis, band intensity of PCR products from immunoprecipitated DNA was compared with that from 0.5% of respective input (Quantity One Analysis Software, Bio-Rad). The amount of chromatin in input and immunoprecipitated samples was also quantified by qPCR using SYBR green PCR master mix and indicated primers for *TTP*, *GAPDH*. Fold enrichment, expressed as IP/Input ratio, was determined from C_t_ values normalized to the untreated control. Data represent the immunoprecipitated/input ratio: 2 ^(Ct input − Ct IP)^.

*Immunofluorescence.* Cells grown in 4 wells slide chambers (Lab-Tek^®^ chamber slides, Sigma Aldrich) were fixed with a 2% paraformaldehyde solution, washed in PBS and permeabilized with 0.02% Triton X-100 in PBS. Cells were blocked with 5% normal goat serum (Jackson ImmunoResearch, West Grove, PA) in PBS containing 1% IgG-free BSA (Jackson ImmunoResearch) and incubated overnight at 4 °C with antibody against Ki-67 (Ab16667, Abcam, 1:200, Cambridge, MA, USA) diluted in blocking solution. Cells were washed twice in PBS and immunostained with a Cy5-conjugated secondary antibody (Ab97077, Abcam) diluted in PBS containing 3% IgG-free BSA and incubated for 1 h at RT. After two washes, cells were counterstained with 1 μg/mL Hoechst 33342 (Sigma Aldrich) and were analyzed by fluorescence microscopy (Evos^®^ FL Cell Imaging System, Advanced Microscopy Group, Life Technology, Mill Creek, WA, USA).

Detection of Dcp1a, TTP and Hedls protein in human colonic tissues was accomplished by using serial sectioned tissue array (CHTN2003CRCprog, Cooperative Human Tissue Network, NCI, Rockville, MD, USA). The array contains 7 cases of normal non-neoplastic colonic mucosa from non-cancer, 7 cases of normal non-neoplastic colonic mucosa from cancer, 7 adenoma < 2 cm in maximum dimension, 7 adenoma >2 cm in maximum dimension, 7 cases of primary invasive adenocarcinoma (stage T1 or T2) and 7 cases of primary invasive adenocarcinoma (stage T3 or T4). Slides were hydrated and antigen-retrieval was performed in citrate buffer in a steam bath for 30 min. Slides were incubated with a monoclonal antibody against anti-Dcp1a (ab57654; Abcam, Cambridge, MA, USA), TTP polyclonal antibody [30], or a polyclonal antibody against Hedls (Cell Signaling) as previously described [30,54]. All primary antibodies were incubated on slides overnight at 4 °C and the staining procedure were performed as described above. Tissues were analyzed by confocal microscopy and P-bodies quantification was performed by particle analysis in binary mode with ImageJ software (National Institute of Health, Bethesda, MD, USA).

*Statistical Analysis.* All data were reported as the mean ± SD. The Student *t*-test was used to determine the statistical significance of the mean for each group. For comparison of EGR1 and TTP expression in normal colonic and tumor tissue from patients, the paired Student *t*-test was used on log2 fold-change values. Differences with *p* < 0.05 (*), *p* < 0.01 (**), *p* < 0.001 (***) were considered significant.

## 5. Conclusions

In summary, our findings show that HDAC inhibitors induce TTP expression in different cancer cells, thus highlighting an epigenetic regulation of TTP in cancer cells. Our results bring new insights into loss of post-transcriptional regulatory circuits during tumor development. Moreover, our data suggest a clinical approach to restore TTP expression in cancer cells with drugs under investigation in clinical trials such as Vorinostat^®^ (SAHA).
